# Electronic Health Literacy and Self-Efficacy Among Primary and Middle School Students in China: A Moderated Mediated Analysis

**DOI:** 10.3390/children11121470

**Published:** 2024-11-30

**Authors:** Lin Tian, Tao Xie, Jinnan Liu, Ying Mao

**Affiliations:** 1 School of Humanities and Social Science, Xi’an Jiaotong University, Xi’an 710049, China; tianlin1994@stu.xjtu.edu.cn; 2 School of Public Policy Administration, Xi’an Jiaotong University, Xi’an 710049, China; xietao2014077049@stu.xjtu.edu.cn (T.X.); kennanliu@stu.xjtu.edu.cn (J.L.)

**Keywords:** eHealth Literacy, general self-efficacy, Chinese primary and middle school students, structural equation modeling, mediating and moderated analysis

## Abstract

Background: The Internet has become a crucial tool for learning, socializing, and entertainment for contemporary minors, and plays an increasingly prominent role in their growth. However, it has been observed that students are often unable to make good judgments about online health information and barely use the Internet to help tackle their health problems. The purpose of this study was to determine the relationship between electronic health literacy (EHL) and general self-efficacy among Chinese primary and middle school students. Methods: A total of 1200 questionnaires were sent out, and 1085 effective questionnaires were received with effective recovery of 90.42%. First, we conducted a confirmatory factor analysis. Second, structural equation modeling (SEM) was used to explore the mechanisms underlying the relationship between EHL and general self-efficacy. Results: The results revealed a significant positive correlation between EHL and self-efficacy. The results of the CFA showed a good fit for the data. The results of SEM showed that the relationship between self-efficacy and health information applications was partially mediated by health information acquisition (β = 0.47, [Bias-Corrected 95%CI: 0.39, 0.59], [Percentile 95%CI: 0.37, 0.58]). Household factors moderated this mediating relationship (β = 0.4, [Bias-Corrected 95%CI: 0.19, 0.61], [Percentile 95%CI: 0.19, 0.61]). Conclusions: Information acquisition was found to play a mediating role between self-efficacy and information application. Household factors moderated the indirect relationship between self-efficacy and information applications through information acquisition.

## 1. Introduction

According to the Statistical Report on Internet Development in China, the number of internet users under the age of 18 in China reached 183 million, and the Internet penetration rate for this group was 95%, considerably higher than that for adults [1]. Among them, 92% of primary school students had access to the Internet and a notable 34% of minors had access to the Internet during their preschool years [1]. The proportion of underage netizens in China who have access to their own Internet devices (mostly on mobile intelligent terminals i.e., smartphones) has reached 83% [1]. The Internet has become a crucial tool for learning, socializing, and entertainment for contemporary minors, and plays an increasingly prominent role in their growth [1].

Health literacy refers to the ability of individuals to obtain and understand health information and make correct health decisions based on the information obtained [2]. Electronic health literacy (EHL) refers to an individual’s ability to obtain, understand, judge, and use health information from electronic resources to tackle their own health problems [3]. This concept involves aspects of health literacy and electronic health [4]. However, it has been observed that students are often unable to make good judgments about online health information and cannot use the Internet to help tackle their health problems [5]. Therefore, the ability to obtain and use health resources on the Internet has become a critical part of student health literacy [6]. Individuals’ access to and use of health information can help improve knowledge, adopt healthy patterns of behavior, rationalize the use of health care services, and reduce health care costs, and is critical to personal and public health outcomes [7,8].

Self-efficacy refers to an individual’s subjective judgment of his ability to exhibit a particular behavior and achieve an expected result [9]. Self-efficacy can significantly affect individuals’ effort levels and willingness to take risks [10]. Self-efficacy describes a person’s confidence in completing their expected work or tasks, so it is a useful measure for understanding students’ ability to obtain electronic health information online [11,12] and has been identified as an important determinant of student health behavior. In other countries, there are clear results demonstrating a relationship between EHL and self-efficacy in adults [12,13,14,15]. Previous research has also found that self-efficacy significantly affects student characteristics. For example, the self-efficacy of students in higher grades was higher than that of students in lower grades [16], and the self-efficacy of rural students was significantly lower than that of their urban peers [17,18]. However, some studies have indicated that the correlation between EHL and self-efficacy is not significant [19], indicating that their relationship remains somewhat controversial. General self-efficacy reflects an individual’s overall belief in their ability to cope with challenges, exert control over their environment, and achieve desired outcomes across a variety of situations [9,10]. General self-efficacy is a broad construct that differs from domain-specific self-efficacy (e.g., health-related or academic self-efficacy) and was chosen for this study due to its foundational role in shaping students’ confidence and resilience. This construct is particularly relevant to understanding how students may apply EHL in various life contexts, as general self-efficacy influences their willingness and ability to engage with and utilize health information effectively.

While studies have explored the relationship between EHL and general self-efficacy in adults, limited research has examined this relationship among primary and secondary school students, especially in the Chinese context. Furthermore, the mechanisms underlying this relationship, such as mediating and moderating factors, remain unclear.

Previous research has identified various demographic factors influencing students’ EHL [20,21,22,23,24]. For example, studies have shown that parental education [21,22,23,24], household registration location [20,24], grade level [20,22], and other household characteristics significantly impact students’ ability to access and use health information. Specifically, higher levels of parental education have been linked to better health literacy outcomes due to greater access to resources and support [21,22,23,24]. Additionally, household registration and grade level have been associated with disparities in access to digital health resources, which are critical for understanding the mechanisms underlying EHL [20,22,24]. This study incorporates these demographic factors to explore their moderating effects on the relationship between self-efficacy and EHL. By doing so, we aim to provide a more nuanced understanding of the contextual factors influencing EHL in primary and middle school students.

This study aims to address these gaps by investigating the relationship between EHL and self-efficacy in Chinese primary and middle school students. Specifically, we use structural equation modeling (SEM) to: Examine the relationship between self-efficacy and EHL. Identify the mediating effects and the moderating effects. This study makes several unique contributions. First, it extends our understanding of the relationship between general self-efficacy and EHL by focusing on a population that has been largely overlooked in previous research. Second, it provides novel insights into the mechanisms through which general self-efficacy influences EHL, offering a more nuanced understanding of these dynamics. Finally, the findings have practical implications for designing targeted interventions to enhance students’ EHL and address health disparities, particularly between urban and rural students. By addressing these objectives, this study contributes to the theoretical and practical advancement of digital health education and policy for younger populations.

## 2. Methods

### 2.1. Participants

#### 2.1.1. Sampling Strategy

A stratified cluster random sampling method was adopted to select primary and secondary school students in Shaanxi of China between June and August 2021. We randomly selected two primary schools, two junior-high and two senior-high. Four classes were selected from each school and all students in the selected classes participated in the survey. We believe this approach ensured a diverse sample, though it is not fully representative of the entire Chinese student population due to geographic constraints.

#### 2.1.2. Inclusion and Exclusion Criteria

Inclusion and Criteria: (1) Primary and Secondary School Students: Participants were required to be enrolled in grades 3–12 of primary, junior high, or senior high schools. These educational stages correspond to students aged approximately 8–18 years, ensuring they represent the intended population for the study. (2) Experience in the use of electronic devices: Participants needed to have prior exposure to and familiarity with electronic devices (e.g., mobile phones, computers) to ensure they could engage with questions related to electronic health literacy and self-efficacy. This criterion was essential for the relevance of the study, as the constructs under investigation heavily rely on digital interactions.

Exclusion Criteria: (1) Students in the Graduation Grade: Students in the final grade of junior high (Grade 9) and senior high (Grade 12) were excluded due to their limited availability for follow-up surveys, as these students typically focus intensively on high-stakes examinations, such as the high school entrance exam or the National College Entrance Exam (Gaokao). (2) Students in Grades 1–2 of Primary School: Younger primary school students (Grades 1–2) were excluded because their cognitive and reading abilities may not be sufficiently developed to independently comprehend and complete the survey.

#### 2.1.3. Participants Characteristics

A total of 1200 questionnaires were distributed, and 1085 valid responses were received, with an effective recovery rate of 90.42%. The association between the questionnaire survey and student’s records and study results was approved by the Biomedical Ethics Committee, School of Medicine, Xi’an Jiaotong University (No. 2021–1525).

As shown in Table 1, among the 1085 students who participated in the survey, 49.72% were male and 51.44% were female; 34.23% were primary school students, 32.20% were junior school students, 31.83% were high school students, 8.13% were an one child, 88.55% were not an one child, 39.48% were city students, and 57.93% were from the countryside; for students’ mothers, 23.15% had primary school education, 30.35% had junior high school education, 19.19% had high school education, 15.22% had university education or above; and for students’ fathers, 10.79% had primary school education, 36.53% had junior high school education, 23.52% had high school education, and 20.11% had university education or above.

### 2.2. Measures

*Electronic health literacy.* The dependent variable in this study was health literacy. The eHEALS, by Norman et al. [3], is the first and currently the most commonly used EHL assessment tool. It primarily measures the self-perception skills of netizens when they seek online health knowledge to apply in real life. EHL was measured using the Chinese version of the eHEALS, which has good reliability and validity among Chinese students [25]. The responses to all questions used a five pointl Likert scale: “I totally do not think so (1)”, “I kind of don’t think so (2)”, “unclear (3)”, “ I think so (4)”, and “ I think so quite positively (5)”. Respondent total health literacy score was calculated using these question responses and ranged from 8–40 points. The higher the score, the higher the level of EHL. In the current study, Cronbach’s alpha for the scale was 0.909.

*Self-Efficacy.* The key independent variable in this study was general self-efficacy, which was measured using the General Self-Efficacy Scale. The scale was developed by Schwarzer et al., and initially had 20 items, it was later reduced to 10 [26]. This study used the Chinese version of the General Self-Efficacy Scale to quantify students’ self-efficacy. This version of the scale offers sufficient reliability and validity to evaluate self-efficacy in a student population [27]. The scale was one-dimensional with no subscales and a 4-point scale grading method was adopted. A response of “1” signifies that the statement is completely incorrect; “2” represents partly correct; “3” indicates that it is basically correct; and “4” signifies that the statement is exactly right. The total score was calculated by adding the scores of all the items. The control variables in this study included gender, age, grade, place of residence, race, one child, and parents’ work style and educational level. In the current study, the Cronbach’s alpha for the scale was 0.877.

*Demographic characteristics.* To explore the differences of demographic characteristics in the relationship of self-efficacy and EHL the following demographic information was also collected: students’ gender, grade, district (urban or rural), number of children in the family (whether one-child or not), father’s education level, and mother’s education level.

### 2.3. Statistical Analysis

We conducted confirmatory factor analysis (CFA) on the measures of the eHEALS and General Self-Efficacy Scale. Where multiple factor structures emerged, SEM was used to explore the mechanisms underlying the relationship between EHL and self-efficacy. Simultaneously, we explored whether regulatory factors affected this internal mechanism. This part of the study was generated using SPSS 22.0 and AMOS 21.

## 3. Results

### 3.1. Confirmatory Factor Analysis

Before testing the association between health literacy and self-efficacy, CFA was used to test the data fit of the measurement. The CFA results showed that eHEALS included two latent variables (health information acquisition and health information application) and six observed variables; self-efficacy showed that the General Self-Efficacy Scale was a latent variable with eight observed variables. The model fit indices showed that the models of the eHEALS and the General Self-Efficacy Scale fit the data well (Table 2). The construct reliability (CR) of the latent variables for both the eHEALS and General Self-Efficacy Scale was greater than 0.8, indicating that all latent factors had high reliability (Table 3). All standardized factor loadings (SFL) were significant (*p* < 0.001, indicating that all latent factors in the current study were adequately represented by their respective indicators (See Table 2 for more details on the CFA).

### 3.2. Mediation Analysis

We expected a significant correlation between self-efficacy and EHL and found that the internal mechanism of the relationship between self-efficacy and EHL, that is, health information acquisition–would mediate the relationship between self-efficacy and health information application. Mediation analyses were conducted using SEM. Table 4 shows the SEM results for the full sample with standardized parameter estimates. Both direct path coefficients from self-efficacy to information application (β = 0.35, *p* < 0.001) and to information acquisition (β = 0.70, *p* < 0.001) were significant. The direct path coefficient from information acquisition to information application (β = 0.68, *p* < 0.001) was significant.

**Figure 1:**
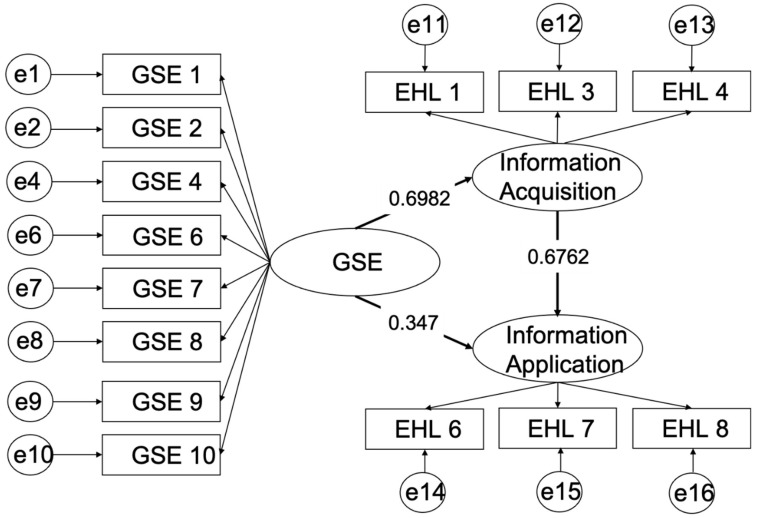
The role of information acquisition between GSE and information application. e1–16: the error terms of the corresponding items.

The bootstrapping method was used to assess the size of the mediation effect and the confidence interval (CI) (Figure 1 and Table 5). In this study, we generated 5000 bootstrapping samples and calculated 95% bootstrap confidence intervals (CIs). Table 4 indicated that the relationship between self-efficacy and health information application was partially mediated by health information acquisition (β = 0.47, [Bias-Corrected 95%CI: 0.37, 0.59], [Percentile 95%CI: 0.37, 0.58]). The 95% CI of both methods did not consist of zero. Table 2 shows that the information acquisition model, as a mediator, revealed a good fit with the data.

### 3.3. Moderated Mediation Analysis

We further explored the differences in gender, grade, household, one child, father’s education, and mother’s education using the mediation model. Table 6 shows whether these factors have a moderating effect on the mediation relationship, that is, whether there is a difference in the mediation relationship between the different groups. Supplementary Tables S1–S6 show the mediating effect analyses for each group. Supplementary Figures S1–S17 show the moderated mediator model. There is a significant difference of indirect effect between city and village dwelling (β = 0.40, [Bias-Corrected 95%CI: 0.19, 0.61], [Percentile 95%CI: 0.19, 0.61]) (Supplementary Table S3, Supplementary Figures S6 and S7). Gender (Supplementary Table S1, Supplementary Figures S1 and S2), grade (Supplementary Table S2, Supplementary Figures S3–S5), one child (Supplementary Table S4, Supplementary Figures S8 and S9), father’s education (Supplementary Table S5, Supplementary Figure S10–S13) and mother’s education (Supplementary Table S6, Supplementary Figures S14–S17) did not have a significant indirect effect. Table 2 shows that these models had a good fit.

## 4. Discussion

The results revealed that self-efficacy and EHL were significantly positively correlated. To the best of our knowledge, this study is the first to demonstrate the relationship between EHL and self-efficacy among Chinese primary and middle school students. By analyzing the relationship between self-efficacy and the two latent variables of EHL, we extend our knowledge of this relationship. Specifically, information acquisition was found to play a mediating role between self-efficacy and information application; the results showed that students with high self-efficacy were more likely to apply health information in real life, and this relationship was mediated by health information acquisition. Household factors moderated the indirect relationship between self-efficacy and information applications through information acquisition.

Self-efficacy was significantly and positively associated with EHL, which is consistent with the results of most previous studies [12,13,14,15]. Holch et al. found that electronic health literacy was significantly and positively correlated with self-efficacy and that self-efficacy was a significant predictor of electronic health literacy scores [12]. One study observed the mediating effect of self-efficacy on the association between electronic health literacy and healthy habits [13]. They found a significant correlation between electronic health literacy and self-efficacy in both groups (a group of adults identified as at risk for chronic back pain and a non-risk group) and revealed a correlation between the main drivers of healthy habits [14]. Pourrazavi et al. [14] recommend self-efficacy as a powerful concept that can play a crucial role in improving older adults’ electronic health literacy. Similarly, Filabadi et al. indicated a positive and statistically significant correlation between electronic health literacy and self-efficacy [25]. Specifically, self-efficacy can enhance students’ acquisition and use of health information. Self-efficacy is an individual’s perception of their own ability to organize and carry out the necessary activities in a certain way [28]. A high level of self-efficacy enables us to take actions in the belief that good results will be achieved. Notably, people with good self-efficacy work within their existing conditions as much as possible to achieve their goals [29]. EHL refers to the ability of individuals to obtain, understand, judge, and use health information from electronic sources to tackle health problems. The essential purpose is to address one’s own health problems using health information. Students with a good sense of self-efficacy are more active in obtaining and applying health-related information from online resources and are more inclined to pay attention to their own health. At the same time, starting from the traditional theory of achievement motivation, self-efficacy can be used as an important motivation for students to acquire and apply health information online. This is also consistent with previous studies, some have found that self-efficacy is significantly and positively correlated with mastery and achievement approach goals [30,31,32]. Mastering health knowledge through online resources and successfully applying this knowledge to achieve health benefits are naturally related to good self-efficacy.

The results of this study revealed a pattern in the relationship between self-efficacy and electronic health, in that students’ ability to access health information mediated the relationship between self-efficacy and the application of health information. Students’ self-efficacy in this area acquired through health information and affect the application of that health information in addressing their own health problems. Students with good self-efficacy had stronger confidence in achieving their goals and stronger abilities to obtain and apply health information. Self-efficacy clearly emerges from students’ individual factors. The acquisition of health information involves a more direct social situation or microenvironment around an individual, which is a situational factor. The application of health information is essentially the occurrence of behavior, which is an environmental factor. From proximal to distal influence and then to final influence, self-efficacy affects the application of health information through its acquisition [33].

We also found that this mechanism is moderated by urban-rural differences. Consistent with other studies [22,23]. We found that urban students’ self-efficacy directly affected the application of health information. The strength of this relationship was higher for rural students; however, the mediating effect of rural students’ access to health information was stronger than that of urban students [34]. On one hand, the environment for urban students to receive health information is more abundant, and health-related knowledge is easily passed on to them [35,36]. Students with a good sense of self-efficacy directly apply acquired health information to address their own health problems. The living environment of rural children makes it more difficult to obtain health-related knowledge and information than that of urban children. At the same time, with the development of information technology and urbanization, the network environment in rural areas has become more convenient, which is not easy to obtain from the living environment. For many students it is convenient to obtain health knowledge online and, once they have obtained this information, they can further leverage their sense of self-efficacy to devote themselves to addressing their own health problems [37].

This study has several limitations. First, this was a cross-sectional study that can one describe the relationship between important variables and cannot draw conclusions about causality. Therefore, future studies where conditions permit, follow-up cohort studies should be conducted to determine temporal changes in the relationship between self-efficacy and EHL. Secondly, as an observational study, while efforts were made to control for potential confounding variables, it is impossible to completely rule out their influence. Sociocultural factors such as internet addiction, cyberbullying, smartphone usage patterns, and broader digitalization initiatives were not explicitly examined. Smartphones, as a key component of digital tools, may play a crucial role in shaping EHL, given their widespread adoption among young people and their integration into educational and social contexts. However, this study focused on overarching EHL and self-efficacy without delving into these potential moderating or confounding factors. Future research could delve deeper into the nuances of smartphone usage, considering differences based on age, educational stage, and access to digital resources provided by schools and communities. Additionally, the rapid advancement of digitalization in China, including the development of smart city services and targeted school programs, warrants further investigation to understand how these initiatives may impact students’ EHL and self-efficacy. Such studies could provide a more comprehensive view of the interplay between individual digital skills, environmental resources, and educational outcomes. Third, the survey data in this study were all based on self-reports of adolescents, and there may have been information bias. Future research could collect data from multiple sources such as adolescent parents, teachers, or peer groups. Finally, the target groups in this study were all from the Shaanxi Province in western China, which has geographical limitations and so the findings cannot be extended to all of China. Future research should collect data from the Eastern and Central regions to test the stability of this relationship.

## 5. Conclusions

To the best of our knowledge, this study is the first to demonstrate the relationship between EHL and self-efficacy among Chinese primary and middle school students. Information acquisition was found to play a mediating role between self-efficacy and information application. Household factors moderated the indirect relationship between self-efficacy and information applications through information acquisition. The discovery of mechanisms for accessing and utilizing health information can help improve individual health outcomes and reduce public health costs.

## Figures and Tables

**Table 1:** The characteristics of primary and middle school Students (N = 1085).

Variables	Characteristic	Variables	Characteristic
Gender		Mother education	
Male	539 (49.72%)	Primary school	251 (23.15%)
Female	541 (51.44%)	Junior high school	329 (30.35%)
Grade		High school	208 (19.19%)
Primary school	371 (34.23%)	University and above	165(15.22%)
Junior high school	349 (32.20%)	Father education	
High school	345 (31.83%)	Primary school	117 (10.79%)
One child		Junior high school	396 (36.53%)
Yes	88 (8.13%)	High school	255 (23.52%)
No	959 (88.55%)	University and above	218 (20.11%)
Place of Residence			
City	428 (39.48%)		
Countryside	628 (57.93%)		

**Table 2:** Summary of Model Fit.

	*X* ^2^	DF	*X*^2^/DF	GFI	AGFI	RMSEA	NFI	IFI
CFA of EHL	42.57	8	5.32	0.98	0.96	0.07	0.98	0.99
CFA of GSE	82.05	20	4.10	0.98	0.96	0.06	0.96	0.97
Mediation of IC	319.18	73	4.37	0.95	0.93	0.06	0.94	0.95
Moderation of Gender	438.60	146	3.00	0.93	0.90	0.05	0.92	0.93
Moderation of Grade	517.00	219	2.36	0.92	0.88	0.04	0.91	0.94
Moderation of Household	434.00	146	2.97	0.93	0.90	0.05	0.92	0.94
Moderation of One child	451.92	146	3.10	0.93	0.91	0.05	0.91	0.94
Moderation of Father Education	629.33	292	2.16	0.91	0.86	0.04	0.88	0.92
Moderation of Mother Education	662.78	292	2.27	0.90	0.86	0.04	0.88	0.93

CFA: Confirmatory factor analysis, EHL: Electronic health literacy, GSE: General self-efficacy, IC: Information acquisition, IA: Information application.

**Table 3:** Confirmatory factor analysis of electronic health literacy and self-efficacy.

Variables	Item	Estimates			Convergent Validity		
		FL	SE	*p*-Value	SFL	SMC	CR
EHL-IC	EHL1	1.00			0.74	0.55	0.85
	EHL3	1.12	0.05	<0.001 ***	0.85	0.73	
	EHL4	1.11	0.05	<0.001 ***	0.83	0.69	
EHL-IA	EHL6	1.00			0.78	0.62	0.86
	EHL7	1.07	0.04	<0.001 ***	0.89	0.79	
	EHL8	0.98	0.04	<0.001 ***	0.77	0.60	
GSE	GSE1	1.00			0.59	0.35	0.84
	GSE2	0.81	0.07	<0.001 ***	0.51	0.26	
	GSE4	0.99	0.07	<0.001 ***	0.61	0.37	
	GSE6	1.06	0.07	<0.001 ***	0.65	0.43	
	GSE7	1.09	0.07	<0.001 ***	0.70	0.49	
	GSE8	1.07	0.07	<0.001 ***	0.68	0.46	
	GSE9	1.08	0.07	<0.001 ***	0.69	0.47	
	GSE10	0.92	0.07	<0.001 ***	0.54	0.29	

*** *p* < 0.001, EHL: Electronic health literacy, GSE: General self-efficacy, IC: Information acquisition, IA: Information application, FL: Factor loading, SFL: Standard factor loading, SE: Standard error, SMC: Squared multiple correlation, CR: Composite reliability, AVE: Average variance extracted.

**Table 4:** The pathway of self-efficacy and electronic health literacy.

	Estimate	S.E.	*p-*Value
GSE→IC	0.70	0.07	<0.001 ***
GSE→IA	0.35	0.07	<0.001 ***
IC→IA	0.68	0.05	<0.001 ***

*** *p* < 0.001, GSE: General self-efficacy, IC: Information acquisition, IA: Information application.

**Table 5:** Testing the Mediation Effect of information acquisition on the relation between electronic health literacy and self-efficacy.

	Point Estimate	Product of Coefficients		Bootstrapping			
				Bias-Corrected 95% CI		Percentile 95% CI	
		SE	Z	Lower	Upper	Lower	Upper
GSE→IA	0.35	0.08	4.33	0.19	0.51	0.19	0.51
GSE→IC→IA	0.47	0.05	8.82	0.37	0.59	0.37	0.58
GSE→IA	0.82	0.09	9.58	0.65	0.99	0.65	0.99

Estimating of 5000 bootstrap sample, GSE: General self-efficacy, IC: Information acquisition, IA: Information application.

**Table 6:** Testing the Moderating Effect of student’s characteristics on the Mediation Effect of information acquisition on the relation between and self-efficacy and electronic health literacy.

		Point Estimate	Bootstrapping					
			Bias-Corrected 95% CI		Percentile 95% CI		*p-*Value	
			Lower	Upper	Lower	Upper	Bias-Corrected	Percentile
Gender	IED of M-F	−0.11	−0.33	0.10	−0.33	0.10	0.31	0.30
Grade	IED of P-J	−0.11	−0.43	0.28	−0.46	0.25	0.61	0.49
	IED of P-H	−0.04	−0.36	0.33	−0.39	0.30	0.87	0.77
	IED of J-H	0.06	−0.23	0.36	−0.22	0.37	0.68	0.65
Household	IED of C-V	0.40	0.19	0.61	0.19	0.61	<0.001 ***	<0.001 ***
One child	IED of Y-N	−0.14	−0.65	0.28	−0.61	0.31	0.51	0.62
Father education	IED of P-J	0.29	−0.20	1.19	−0.24	1.11	0.26	0.33
	IED of P-H	0.43	−0.07	1.32	−0.09	1.24	0.10	0.13
	IED of P-U	0.21	−0.34	1.07	−0.37	1.02	0.47	0.53
	IED of J-H	0.14	−0.13	0.42	−0.12	0.43	0.33	0.30
	IED of J-U	−0.08	−0.43	0.20	−0.40	0.23	0.52	0.63
	IED of H-U	−0.22	−0.59	0.10	−0.58	0.11	0.18	0.19
Mother education	IED of P-J	−0.09	−0.38	0.24	−0.39	0.23	0.62	0.55
	IED of P-H	−0.05	−0.37	0.26	−0.35	0.28	0.70	0.79
	IED of P-U	−0.05	−0.48	0.32	−0.45	0.35	0.75	0.87
	IED of J-H	0.04	−0.28	0.34	−0.26	0.37	0.87	0.74
	IED of J-U	0.04	−0.41	0.38	−0.35	0.42	0.94	0.75
	IED of H-U	0.00	−0.41	0.36	−0.39	0.38	0.98	0.98

Estimating of 5000 bootstrap sample, *** *p* < 0.001, TS: Two-tailed significance, IED: Indirect effects difference, M-F: Male between female, C-V: City and village, Y-N: Yes and no, P-J: Primary school and junior high school, P-H: Primary school and high school, P-U: Primary school and university, J-H: Junior high school and high school, J-U: Junior high school and university, H-U: High school and university.

## Data Availability

Data are contained within the article.

## Supplementary Materials

The following supporting information can be downloaded at: https://www.mdpi.com/article/10.3390/children11121470/s1, Table S1: The mediating role of information acquisition between self-efficacy and information application (gender); Supplementary Table S2 The mediating role of information acquisition between self-efficacy and information application (grade). Supplementary Table S3 The mediating role of information acquisition between self-efficacy and information application (household). Supplementary Table S4 The mediating role of information acquisition between self-efficacy and information application (only child). Supplementary Table S5 The mediating role of information acquisition between self-efficacy and information application (father education). Supplementary Table S6 The mediating role of information acquisition between self-efficacy and information application (mother education); Supplementary Figure S1 The mediating role of information acquisition between self-efficacy and information application (Male); Supplementary Figure S2 The mediating role of information acquisition between self-efficacy and information application (Female) Supplementary Figure S3 The mediating role of information acquisition between self-efficacy and information application (Primary school); Supplementary Figure S4 The mediating role of information acquisition between self-efficacy and information application (Junior school); Supplementary Figure S5 The mediating role of information acquisition between self-efficacy and information application (High school); Supplementary Figure S6 The mediating role of information acquisition between self-efficacy and information application (City); Supplementary Figure S7 The mediating role of information acquisition between self-efficacy and information application (Countryside); Supplementary Figure S8 The mediating role of information acquisition between self-efficacy and information application (Yes); Supplementary Figure S9 The mediating role of information acquisition between self-efficacy and information application (No); Supplementary Figure S10 The mediating role of information acquisition between self-efficacy and information application (Primary school of father); Supplementary Figure S11 The mediating role of information acquisition between self-efficacy and information application (Junior school of father); Supplementary Figure S12 The mediating role of information acquisition between self-efficacy and information application (High school of father); Supplementary Figure S13 The mediating role of information acquisition between self-efficacy and information application (University of father); Supplementary Figure S14 The mediating role of information acquisition between self-efficacy and information application (Primary school of mother); Supplementary Figure S15 The mediating role of information acquisition between self-efficacy and information application (Junior school of mother); Supplementary Figure S16 The mediating role of information acquisition between self-efficacy and information application (High school of mother); Supplementary Figure S17 The mediating role of information acquisition between self-efficacy and information application (University of mother).
