# Supplementary material for: Electronic Health Literacy and Self-Efficacy Among Primary and Middle School Students in China: A Moderated Mediated Analysis
Source: Children (Basel). 2024 Nov 30;11(12):1470. doi: 10.3390/children11121470 (PMC11674169; doi:10.3390/children11121470)
Supplement: Supplementary file 1 [file children-11-01470-s001.zip › children-3297777-supplementary.pdf]

Table:

Supplementary Table S1 The mediating role of information acquisition between self-efficacy and information application (gender).

Supplementary Table S2 The mediating role of information acquisition between self-efficacy and information application (grade).

Supplementary Table S3 The mediating role of information acquisition between self-efficacy and information application (household).

Supplementary Table S4 The mediating role of information acquisition between self-efficacy and information application (only child).

Supplementary Table S5 The mediating role of information acquisition between self-efficacy and information application (father education).

Supplementary Table S6 The mediating role of information acquisition between self-efficacy and information application (mother education).

Figure:

Supplementary Figure S1 The mediating role of information acquisition between self-efficacy and information application (Male)

Supplementary Figure S2 The mediating role of information acquisition between self-efficacy and information application (Female)

Supplementary Figure S3 The mediating role of information acquisition between self-efficacy and information application (Primary school)

Supplementary Figure S4 The mediating role of information acquisition between self-efficacy and information application (Junior school)

Supplementary Figure S5 The mediating role of information acquisition between self-efficacy and information application (High school).

Supplementary Figure S6 The mediating role of information acquisition between self-efficacy and information application (City).

Supplementary Figure S7 The mediating role of information acquisition between self-efficacy and information application (Countryside).

Supplementary Figure S8 The mediating role of information acquisition between self-efficacy and information application (Yes).

Supplementary Figure S9 The mediating role of information acquisition between self-efficacy and information application (No).

Supplementary Figure S10 The mediating role of information acquisition between self-efficacy and information application (Primary school of father).

Supplementary Figure S11 The mediating role of information acquisition between self-efficacy and information application (Junior school of father).

Supplementary Figure S12 The mediating role of information acquisition between

self-efficacy and information application (High school of father).

Supplementary Figure S13 The mediating role of information acquisition between self-efficacy and information application (University of father)

Supplementary Figure S14 The mediating role of information acquisition between self-efficacy and information application (Primary school of mother).

Supplementary Figure S15 The mediating role of information acquisition between self-efficacy and information application (Junior school of mother).

Supplementary Figure S16 The mediating role of information acquisition between self-efficacy and information application (High school of mother).

Supplementary Figure S17 The mediating role of information acquisition between self-efficacy and information application (University of mother).

Supplementary Table S1 The mediating role of information acquisition between self-efficacy and information application (gender).

Gender		Point estimate	Product of coefficients		Bootstrapping			
					Bias-Corrected 95% CI		Percentile 95% CI	
			SE	Z	Lower	Upper	Lower	Upper
Male	*Direct effects*	0.3188	0.12	2.6567	0.1032	0.5667	0.0991	0.5635
	Indirect effects	0.5237	0.0785	6.6713	0.3926	0.703	0.3754	0.6875
	Total effects	0.8425	0.121	6.9628	0.614	1.0916	0.6086	1.0858
Female	*Direct effects*	0.3987	0.1154	3.4549	0.1865	0.6404	0.1855	0.6395
	Indirect effects	0.4149	0.0786	5.2786	0.2745	0.5847	0.2615	0.5675
	Total effects	0.8136	0.1271	6.4013	0.5838	1.0751	0.569	1.0624

Note: Estimating of 5,000 bootstrap sample, *p < 0.05, **p < 0.01, ***p < 0.001, TS=Two-tailed significance

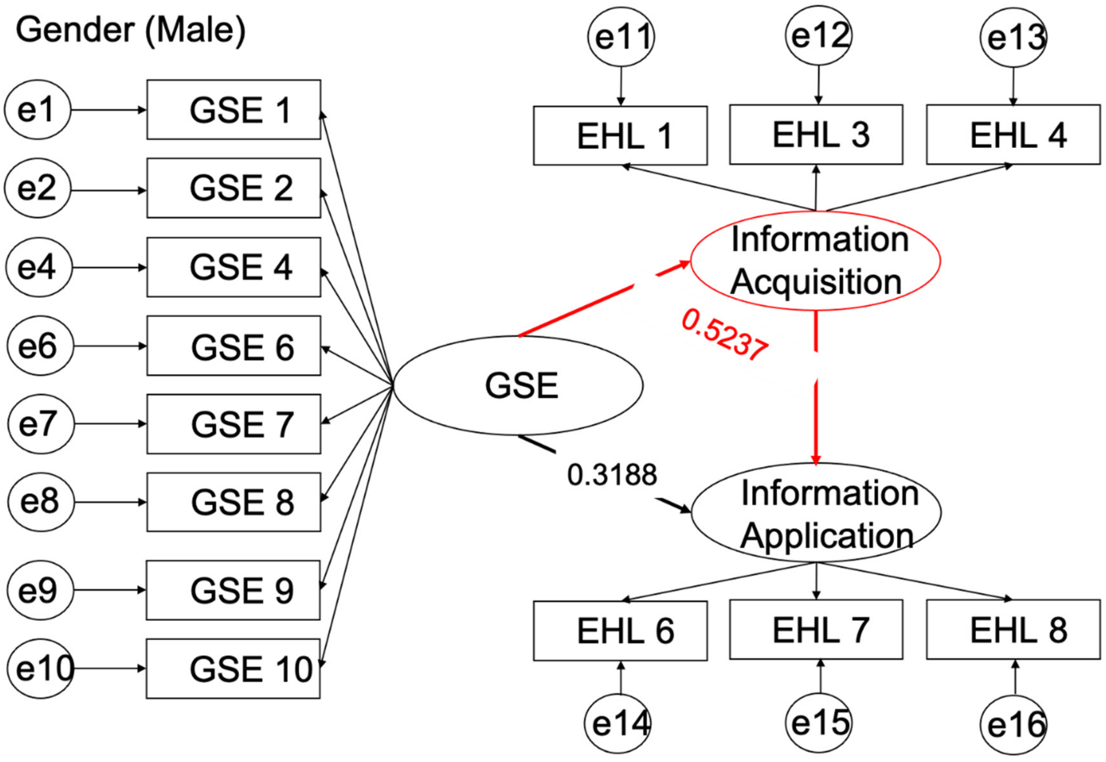

Supplementary Figure S1 The mediating role of information acquisition between self-efficacy and information application (male)

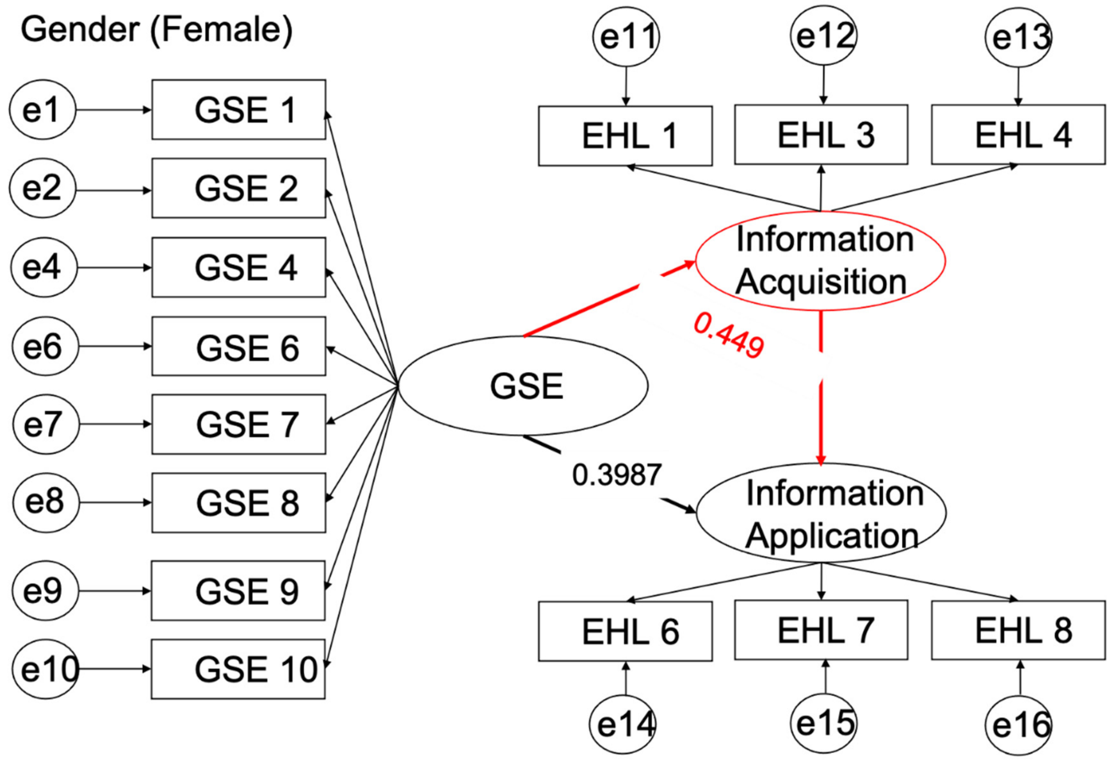

Supplementary Figure S2 The mediating role of information acquisition between self-efficacy and information application (female)

Supplementary Table S2 The mediating role of information acquisition between self-efficacy and information application (grade).

Grade		Point estimate	Product of coefficients		Bootstrapping			
					Bias-Corrected 95% CI		Percentile 95% CI	
			SE	Z	Lower	Upper	Lower	Upper
Primary school	*Direct effects*	1.1064	0.4909	2.2538	0.3819	2.2378	0.4012	2.3005
	Indirect effects	0.4692	0.1428	3.2857	0.2361	0.8322	0.1968	0.76
	Total effects	1.5756	0.4872	3.2340	0.8792	2.7812	0.8731	2.7568
Junior school	*Direct effects*	0.4342	0.1233	3.5215	0.2156	0.7017	0.2155	0.7016
	Indirect effects	0.5744	0.1074	5.3482	0.3864	0.8107	0.3794	0.8009
	Total effects	1.0085	0.1524	6.6175	0.733	1.3385	0.7274	1.3338
High school	*Direct effects*	0.2597	0.0977	2.6581	0.082	0.4629	0.078	0.458
	Indirect effects	0.5122	0.1007	5.0864	0.3255	0.7269	0.314	0.7134
	Total effects	0.7719	0.125	6.1752	0.5182	1.0019	0.5099	0.9987

Note: Estimating of 5,000 bootstrap sample, $*p < 0.05$, $**p < 0.01$, $***p < 0.001$, TS=Two-tailed significance

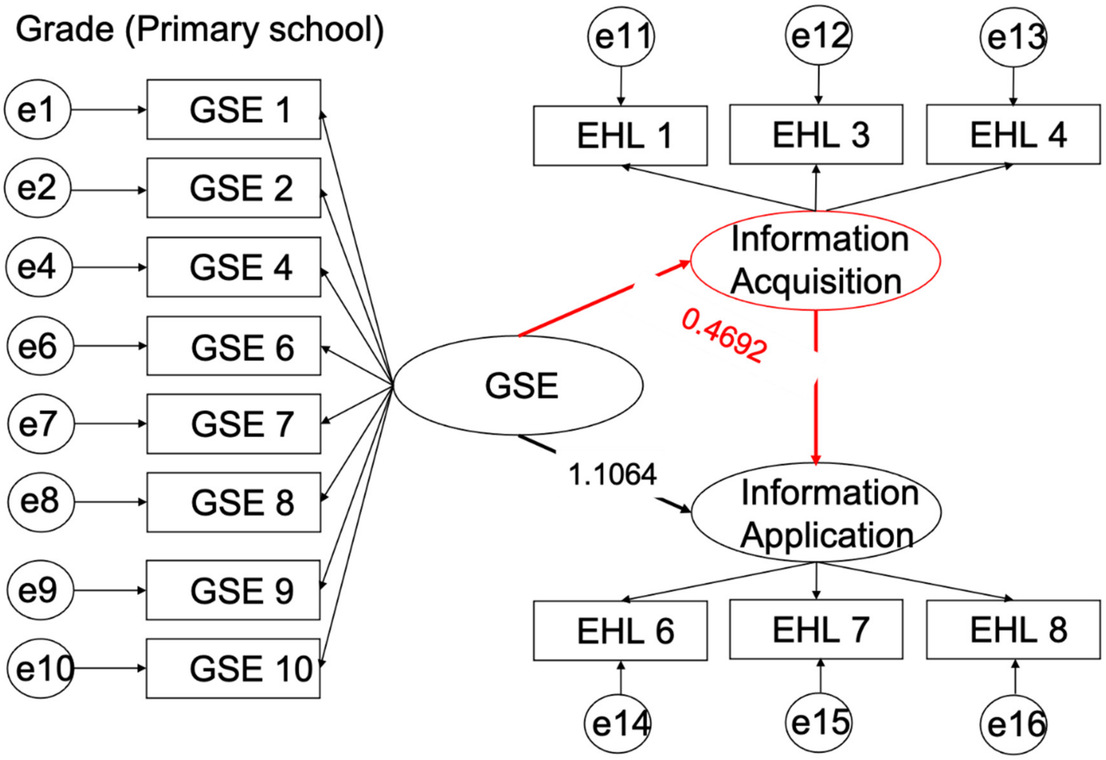

Supplementary Figure S3 The mediating role of information acquisition between

self-efficacy and information application (Primary school)

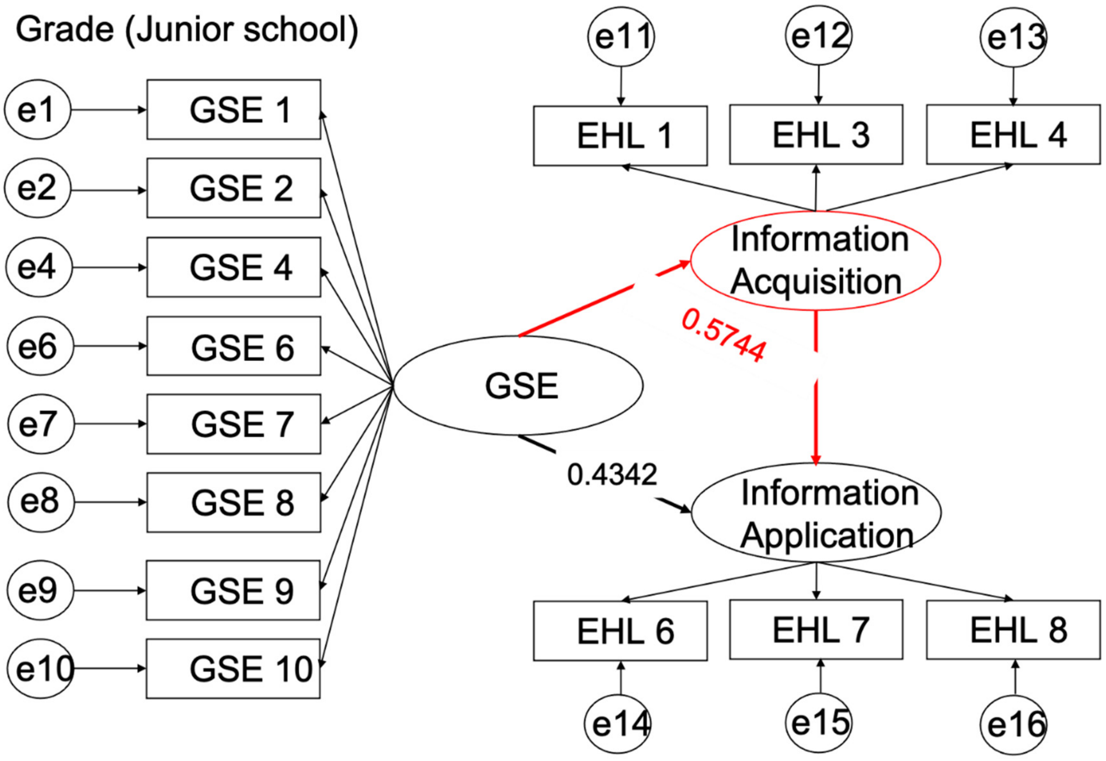

Supplementary Figure S4 The mediating role of information acquisition between self-efficacy and information application (Junior school)

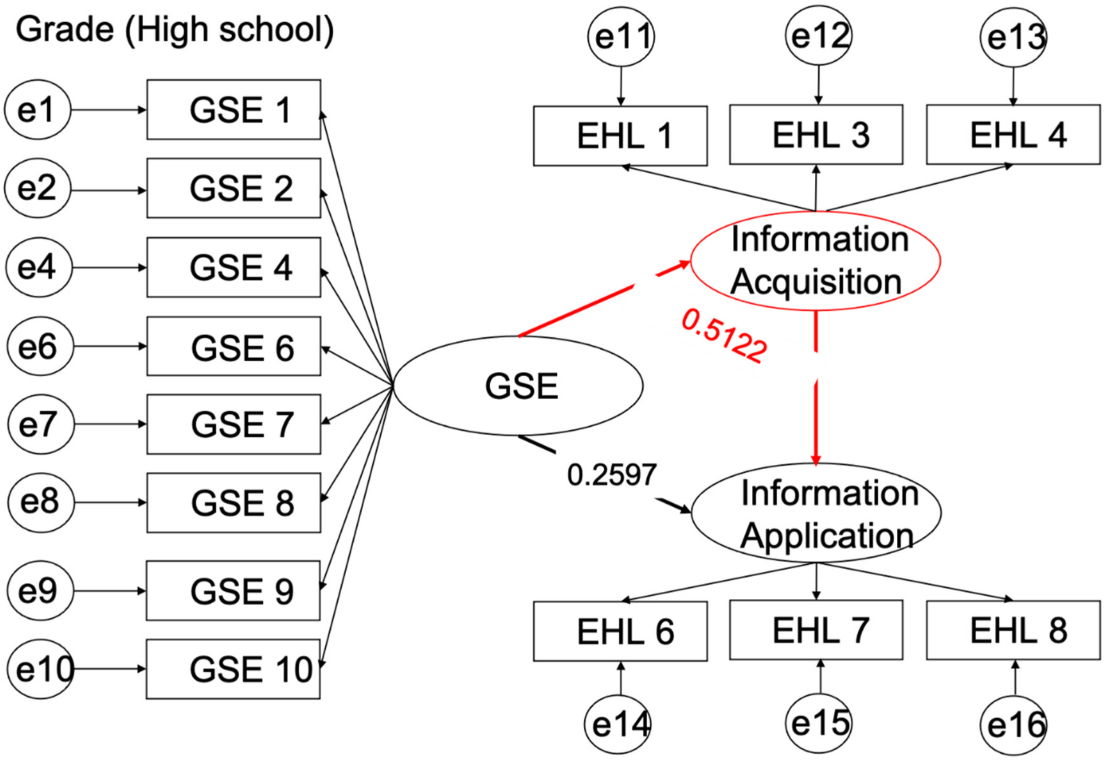

Supplementary Figure S5 The mediating role of information acquisition between

self-efficacy and information application (High school).

Supplementary Table S3 The mediating role of information acquisition between self-efficacy and information application (household).

Household		Point estimate	Product of coefficients		Bootstrapping			
					Bias-Corrected 95% CI		Percentile 95% CI	
			SE	Z	Lower	Upper	Lower	Upper
City	*Direct effects*	0.433	0.1371	3.1583	0.1803	0.7204	0.1836	0.7255
	Indirect effects	0.2538	0.0737	3.4437	0.124	0.4129	0.1163	0.4013
	Total effects	0.6868	0.1461	4.7009	0.4075	0.9866	0.409	0.99
Countryside	*Direct effects*	0.2574	0.0955	2.6953	0.0773	0.4526	0.0759	0.4514
	Indirect effects	0.6518	0.0776	8.3995	0.5132	0.8187	0.5032	0.8114
	Total effects	0.9092	0.1038	8.7592	0.7115	1.1248	0.7076	1.1197

Note: Estimating of 5,000 bootstrap sample, *p < 0.05, **p < 0.01, ***p < 0.001, TS=Two-tailed significance

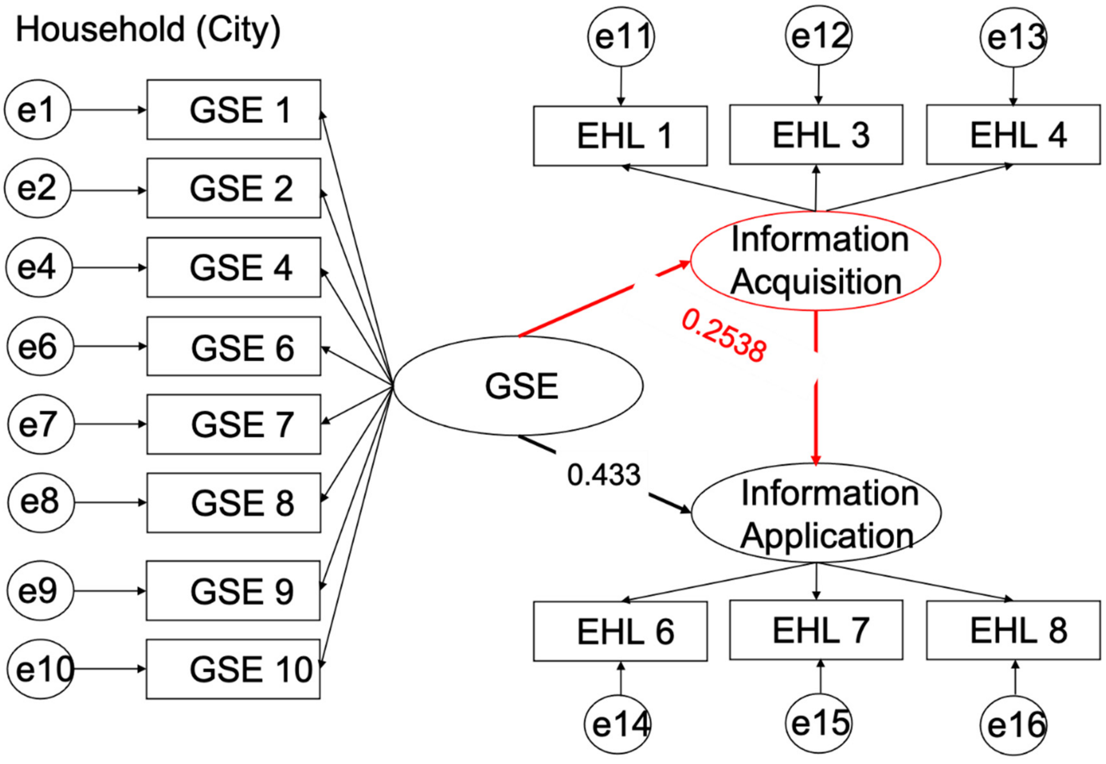

Supplementary Figure S6 The mediating role of information acquisition between self-efficacy and information application (City).

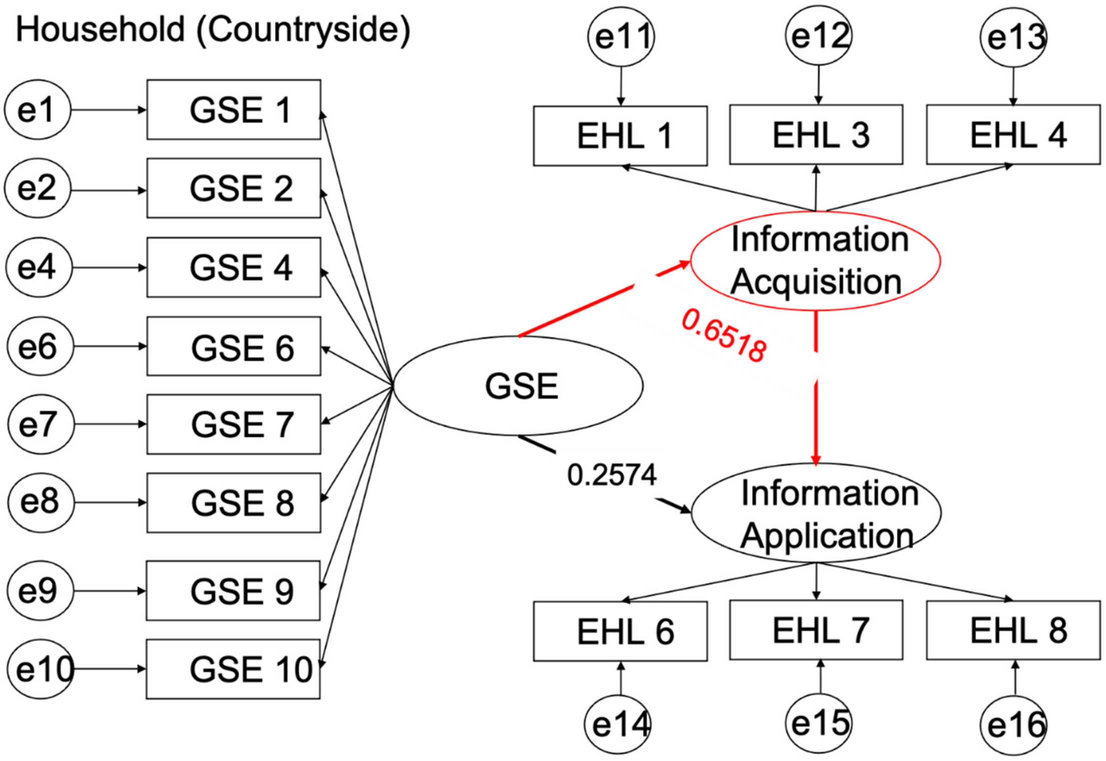

Supplementary Figure S7 The mediating role of information acquisition between self-efficacy and information application (Countryside).

Supplementary Table S4 The mediating role of information acquisition between self-efficacy and information application (only child).

Only child		Point estimate	Product of coefficients		Bootstrapping			
					Bias-Corrected 95% CI		Percentile 95% CI	
			SE	Z	Lower	Upper	Lower	Upper
Yes	*Direct effects*	0.5585	0.4255	1.3126	-0.0474	1.5688	-0.0409	1.5946
	Indirect effects	0.6065	0.2262	2.6813	0.2074	1.1176	0.168	1.0505
	Total effects	1.165	0.3667	3.1770	0.6595	2.1237	0.6417	2.085
No	*Direct effects*	0.3168	0.0812	3.9015	0.1648	0.4804	0.1675	0.4844
	Indirect effects	0.466	0.0568	8.2042	0.3676	0.5901	0.3586	0.5808
	Total effects	0.7828	0.089	8.7955	0.6164	0.9652	0.6164	0.9651

Note: Estimating of 5,000 bootstrap sample, *p < 0.05, **p < 0.01, ***p < 0.001, TS=Two-tailed significance

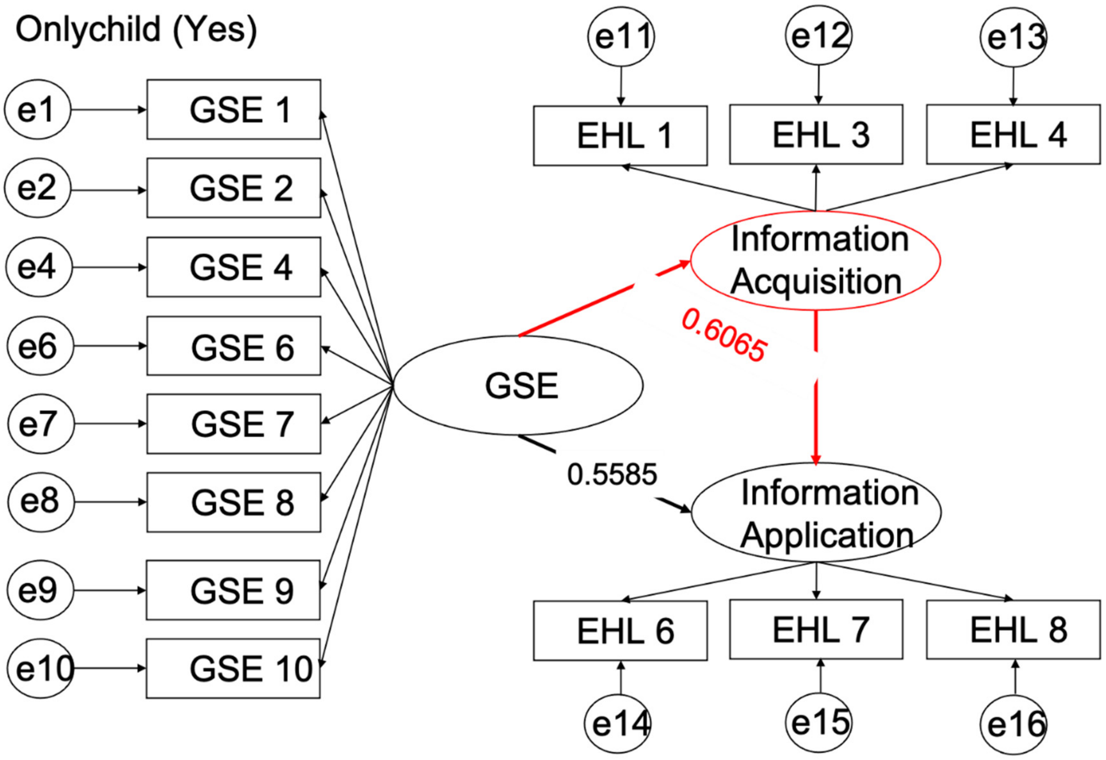

Supplementary Figure S8 The mediating role of information acquisition between self-efficacy and information application (Yes).

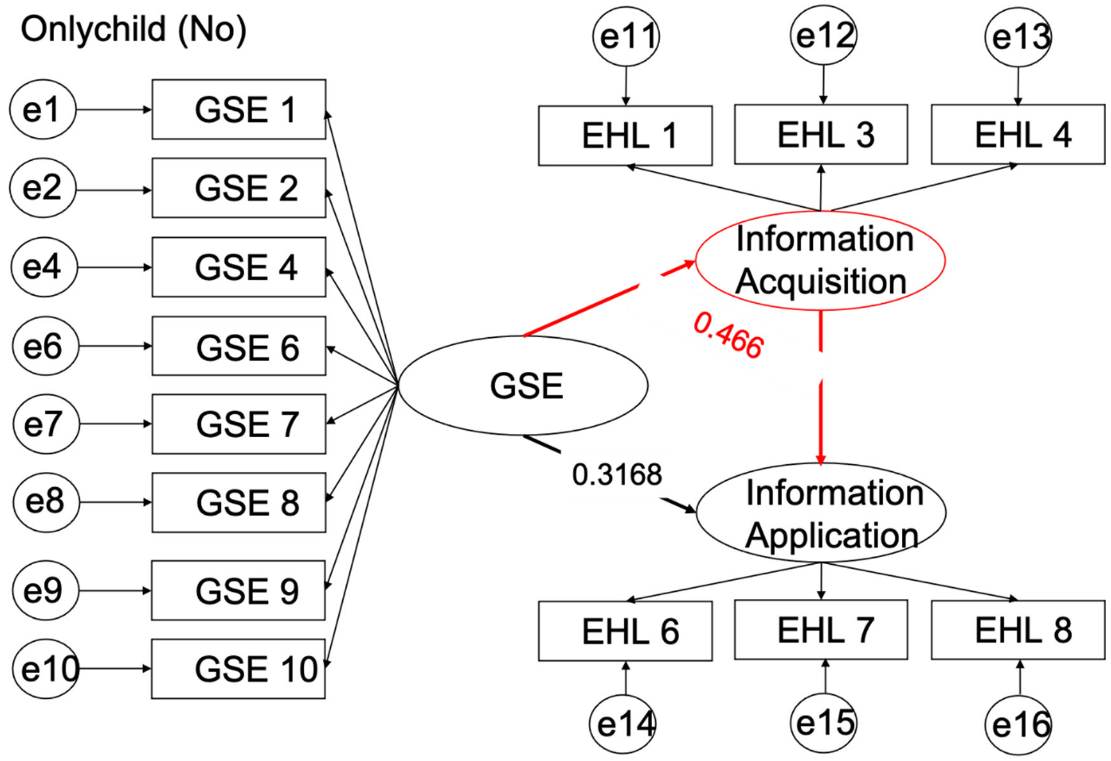

Supplementary Figure S9 The mediating role of information acquisition between self-efficacy and information application (No).

Supplementary Table S5 The mediating role of information acquisition between self-efficacy and information application (father education).

Father Education		Point estimate	Product of coefficients		Bootstrapping			
					Bias-Corrected 95% CI		Percentile 95% CI	
			SE	Z	Lower	Upper	Lower	Upper
Primary school	*Direct effects*	0.4283	0.3726	1.1495	-0.1894	1.2527	-0.1907	1.2518
	Indirect effects	0.7514	0.3288	2.2853	0.3011	1.6643	0.2586	1.5359
	Total effects	1.1798	0.4227	2.7911	0.5505	2.2296	0.536	2.1622
Junior school	*Direct effects*	0.3888	0.1104	3.5217	0.1901	0.6212	0.1879	0.6186
	Indirect effects	0.4628	0.0852	5.4319	0.3175	0.6663	0.3005	0.6406
	Total effects	0.8516	0.1319	6.4564	0.6109	1.119	0.6086	1.1145
High school	*Direct effects*	0.1382	0.1499	0.9219	-0.1415	0.4439	-0.1421	0.4438
	Indirect effects	0.3209	0.1126	2.8499	0.1019	0.5501	0.0843	0.5304
	Total effects	0.4592	0.1764	2.6032	0.1219	0.8206	0.1033	0.8028
University	*Direct effects*	0.5409	0.2479	2.1819	0.1081	1.077	0.1329	1.1138
	Indirect effects	0.5459	0.1344	4.0618	0.3202	0.8681	0.29	0.8177
	Total effects	1.0867	0.2249	4.8319	0.712	1.6193	0.7065	1.6072

Note: Estimating of 5,000 bootstrap sample, *p < 0.05, **p < 0.01, ***p < 0.001, TS=Two-tailed significance

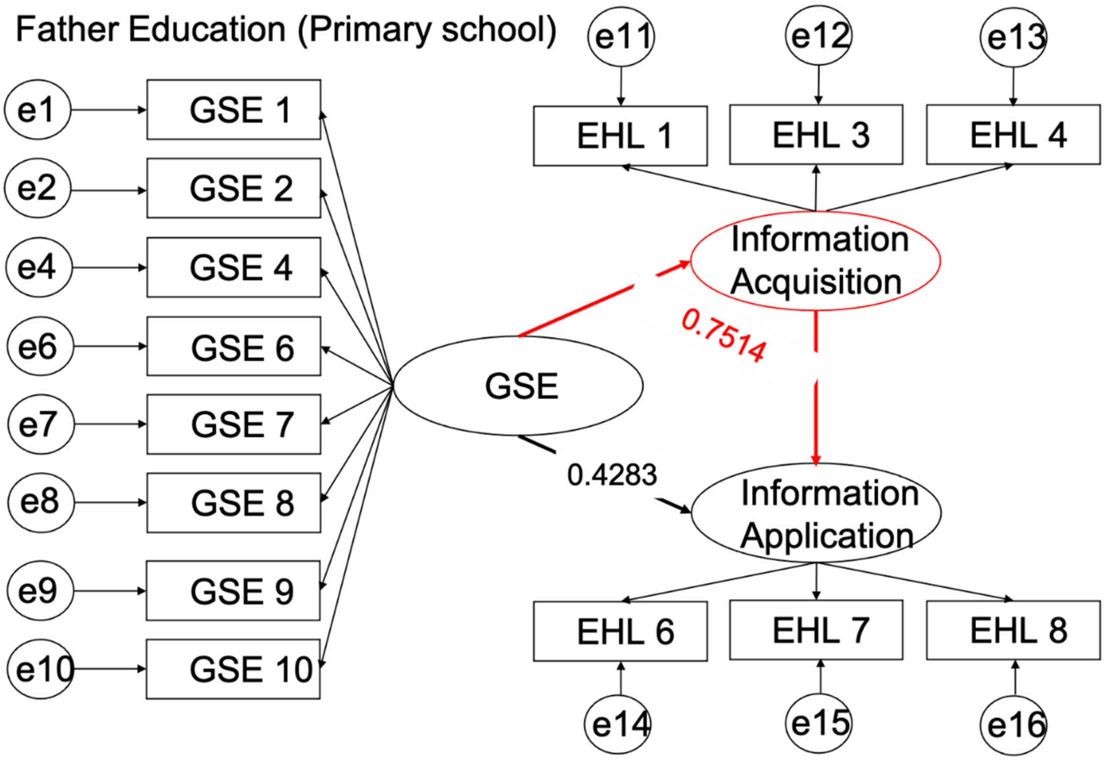

Supplementary Figure S10 The mediating role of information acquisition between self-efficacy and information application (Primary school of father).

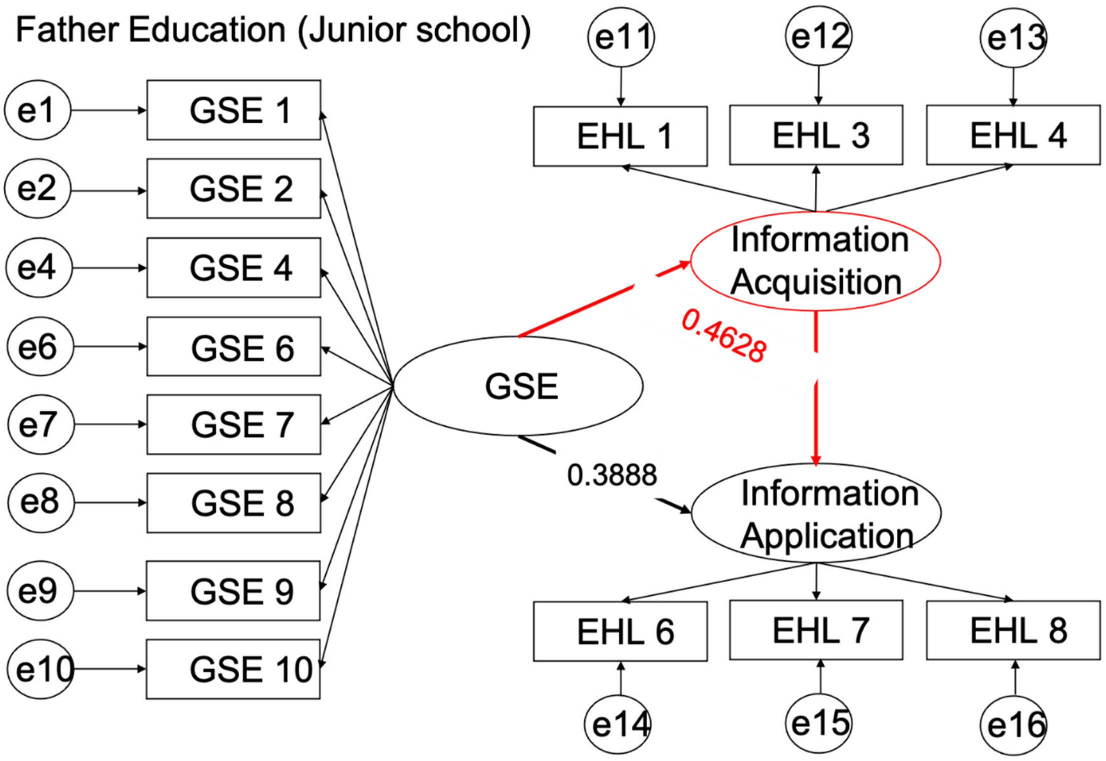

Supplementary Figure S11 The mediating role of information acquisition between self-efficacy and information application (Junior school of father).

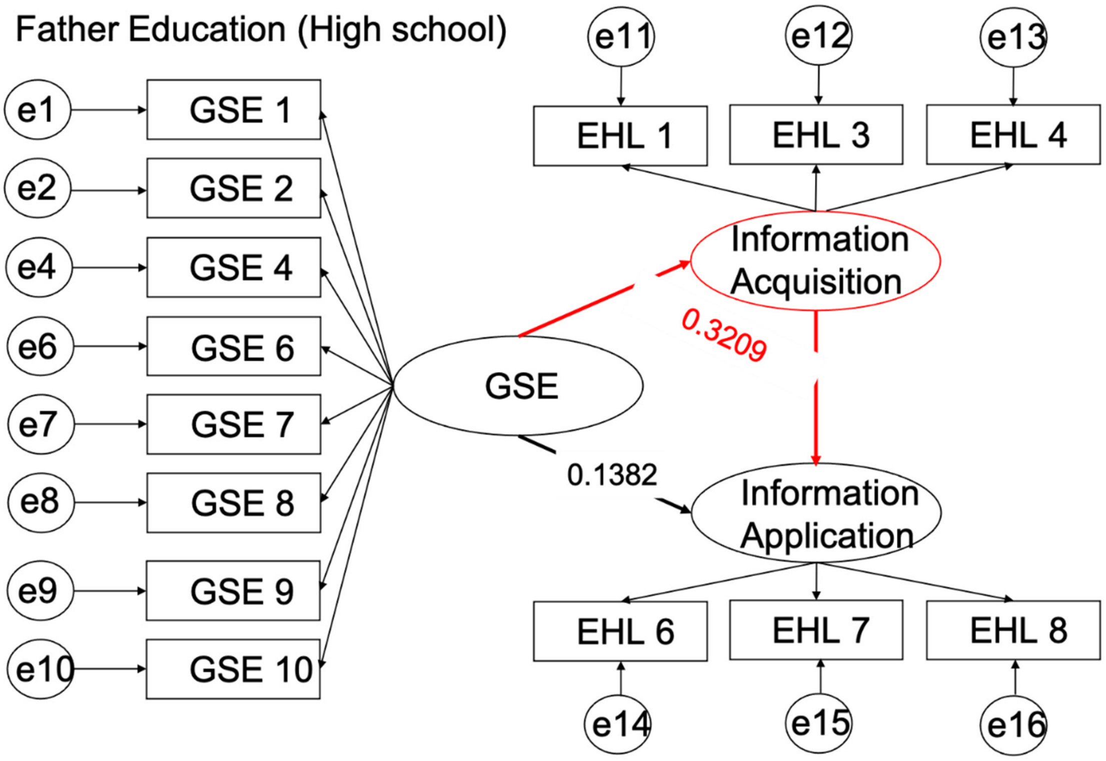

Supplementary Figure S12 The mediating role of information acquisition between self-efficacy and information application (High school of father).

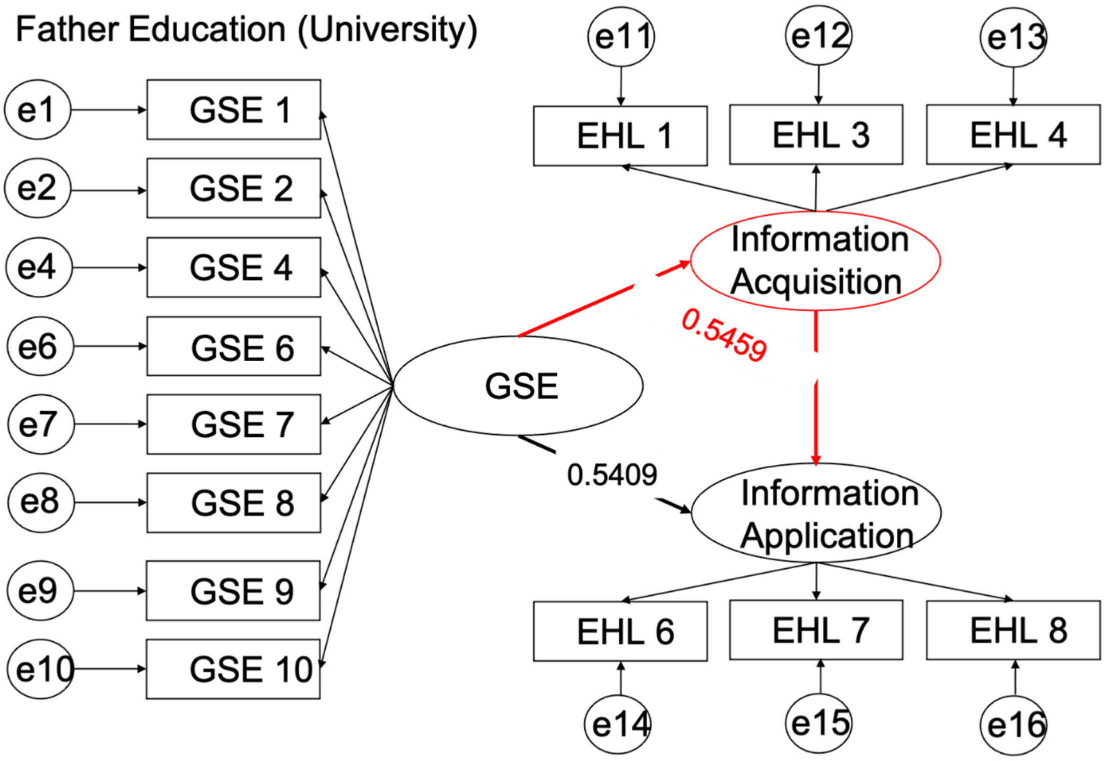

Supplementary Figure S13 The mediating role of information acquisition between self-efficacy and information application (University of father)

Supplementary Table S6 The mediating role of information acquisition between self-efficacy and information application (mother education).

Mother Education		Point estimate	Product of coefficients		Bootstrapping			
					Bias-Corrected 95% CI		Percentile 95% CI	
			SE	Z	Lower	Upper	Lower	Upper
Primary school	*Direct effects*	0.4434	0.1714	2.5869	0.1512	0.8218	0.1379	0.7997
	Indirect effects	0.4219	0.1114	3.7873	0.236	0.6868	0.2194	0.659
	Total effects	0.8653	0.1574	5.4975	0.5938	1.2174	0.5709	1.1942
Junior school	*Direct effects*	0.1901	0.1356	1.4019	-0.0817	0.452	-0.0654	0.4738
	Indirect effects	0.5112	0.1124	4.5480	0.3064	0.7496	0.2975	0.7353
	Total effects	0.7013	0.1734	4.0444	0.363	1.0504	0.3743	1.0629
High school	*Direct effects*	0.3381	0.1596	2.1184	0.0846	0.703	0.0915	0.7129
	Indirect effects	0.4714	0.1108	4.2545	0.2754	0.7118	0.2436	0.6785
	Total effects	0.8095	0.1778	4.5529	0.4999	1.2023	0.4948	1.194
University	*Direct effects*	0.675	0.3202	2.1081	0.1381	1.3555	0.1611	1.386
	Indirect effects	0.4711	0.1624	2.9009	0.2035	0.8672	0.1573	0.8054
	Total effects	1.1461	0.2916	3.9304	0.6965	1.8304	0.6737	1.7902

Note: Estimating of 5,000 bootstrap sample, *p < 0.05, **p < 0.01, ***p < 0.001, TS=Two-tailed significance

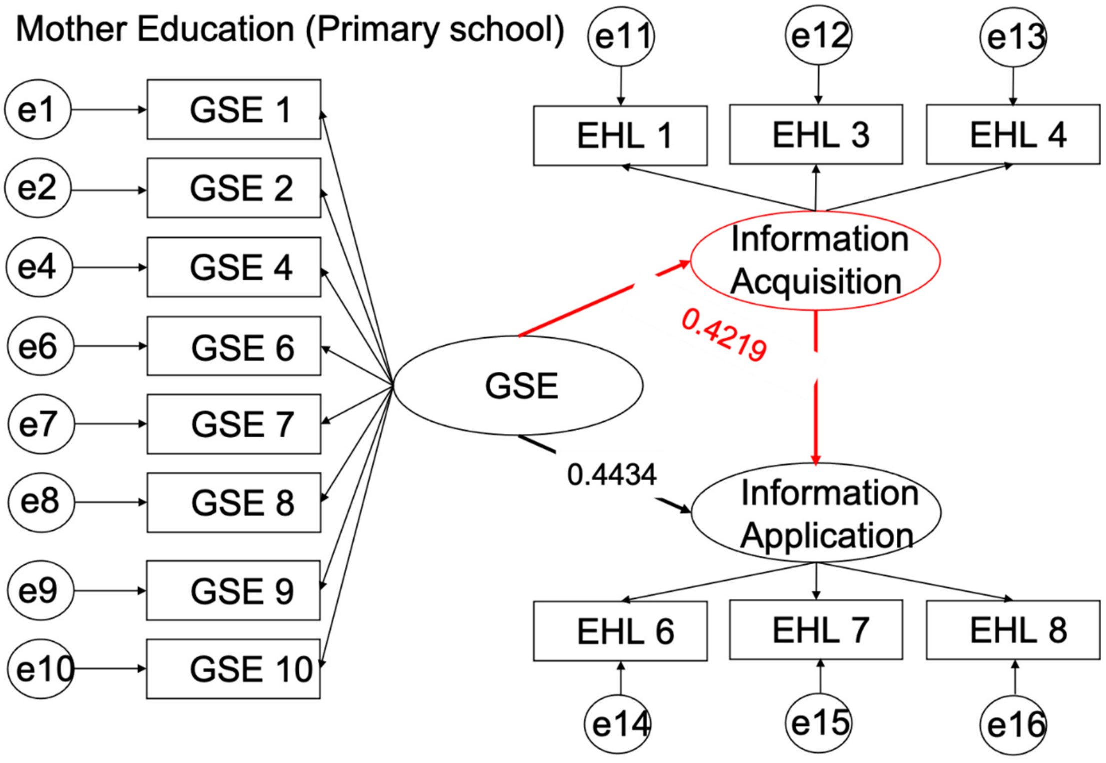

Supplementary Figure S14 The mediating role of information acquisition between self-efficacy and information application (Primary school of mother).

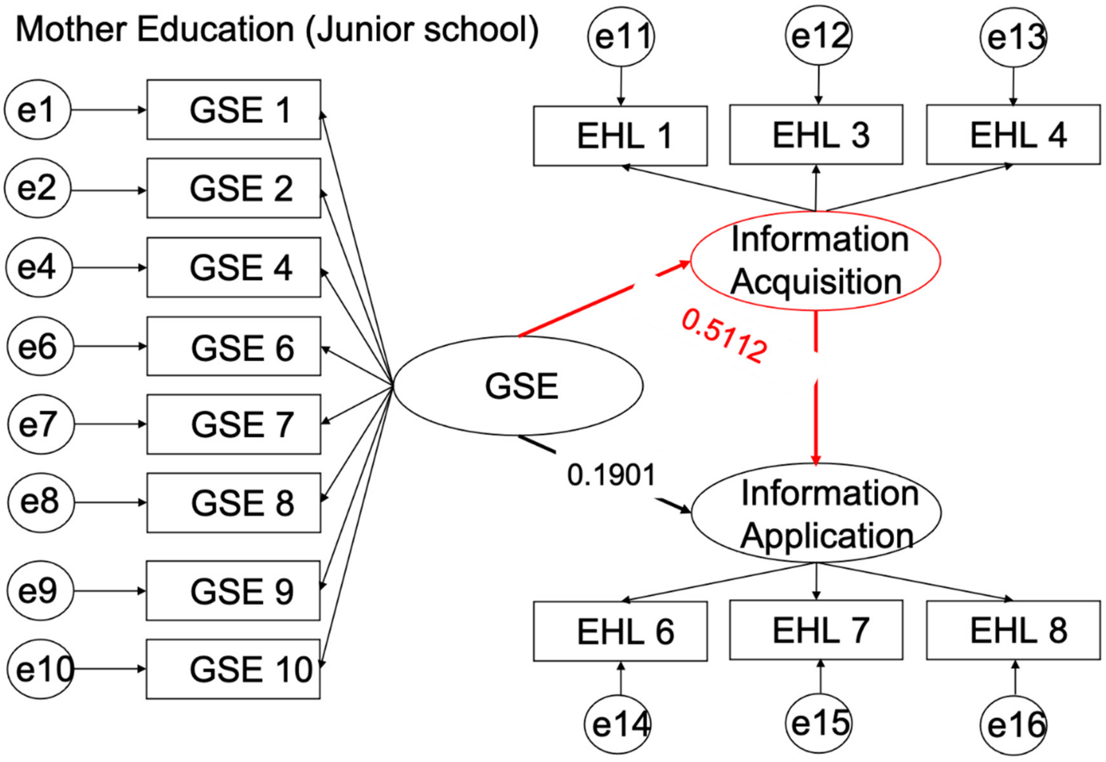

Supplementary Figure S15 The mediating role of information acquisition between self-efficacy and information application (Junior school of mother).

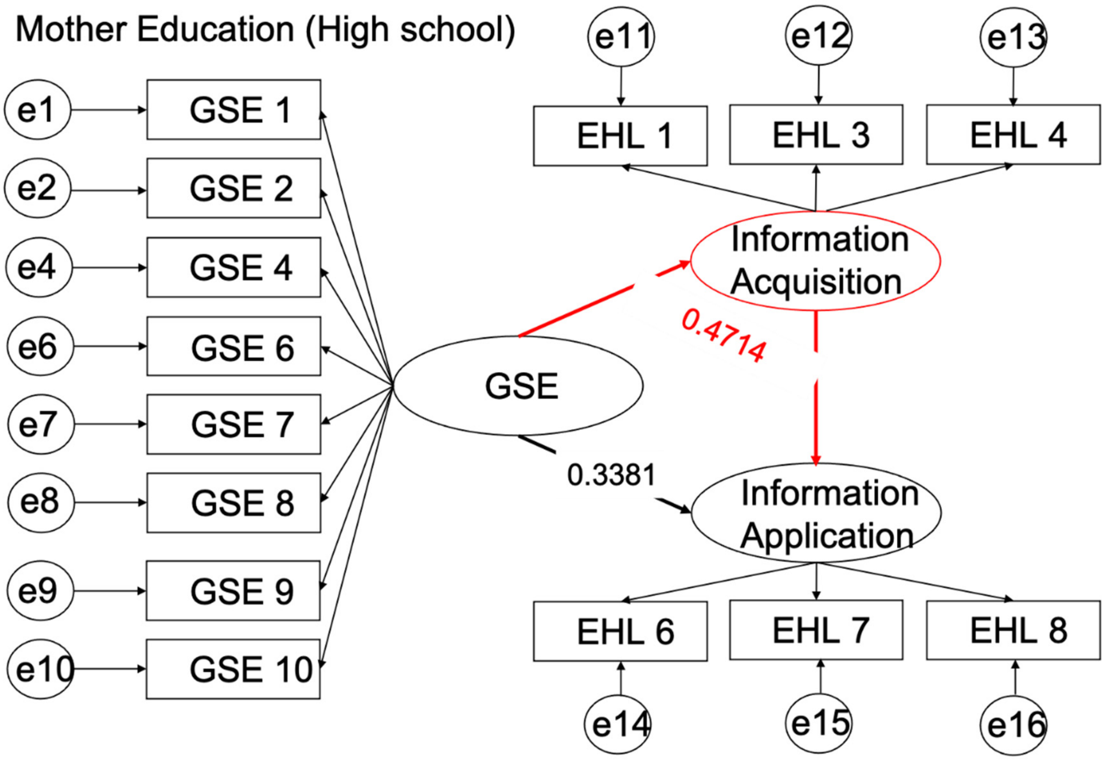

Supplementary Figure S16 The mediating role of information acquisition between self-efficacy and information application (High school of mother).

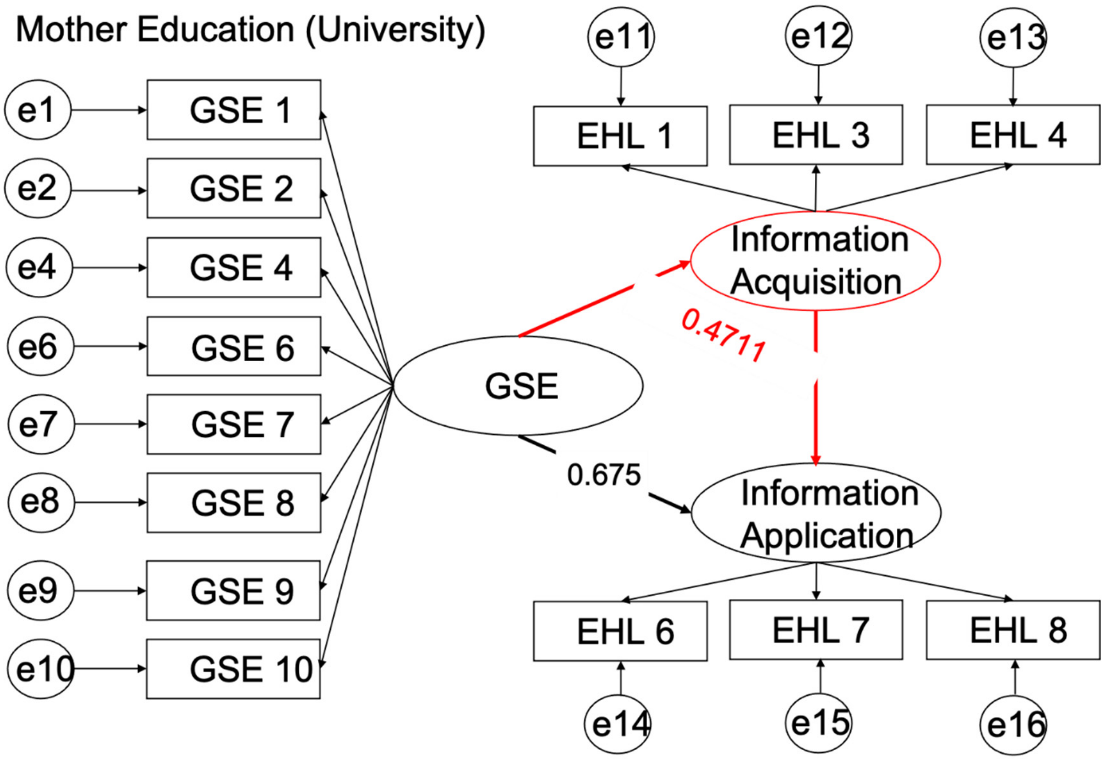

Supplementary Figure S17 The mediating role of information acquisition between self-efficacy and information application (University of mother).
